# CoSAG-nf: a scalable nextflow pipeline for co-assembly, optimization, and interactive visualization of high-throughput single-cell genomes

**DOI:** 10.1093/bioinformatics/btag671

**Published:** 2026-09-17

**Authors:** Linfeng Xu, Zhe-Xue Quan

**Affiliations:** Fudan Microbiome Center, School of Life Sciences, Fudan University, Yangpu District, Shanghai 200438, China; Fudan Microbiome Center, School of Life Sciences, Fudan University, Yangpu District, Shanghai 200438, China

## Abstract

**Motivation:**

Single-cell amplified genomes (SAGs) are crucial for resolving intra-population microbial heterogeneity and accurately understanding the metabolic potential of microbial dark matter populations. However, SAGs generated through multiple displacement amplification (MDA) of genomic DNA from single cells with single-copy chromosomes are highly fragmented and prone to contamination, severely hindering high-quality genome reconstruction and functional analysis, which greatly limits their scientific utility. Co-assembly of related SAGs can substantially improve genome quality, but to our knowledge no automated pipeline exists for high-throughput processing, forcing manual implementation of complex workflows that scale poorly to modern dataset sizes.

**Results:**

We present CoSAG-nf, an automated high-throughput co-assembly and optimization pipeline for SAGs, implemented following the nf-core framework standards. The pipeline performs alignment-free clustering using sourmash MinHash signatures, then employs iterative tetranucleotide frequency profiling to identify and exclude outlier SAGs from co-assembly groups. CheckM2 quality assessment guides dynamic selection of optimal SAG combinations to optimize genome completeness and minimize contamination. Fully containerized, CoSAG-nf ensures reproducibility and scalability for the high-throughput processing of large-scale SAG datasets across diverse computing environments, including HPC and cloud platforms. The pipeline generates comprehensive HTML reports with quality metrics and taxonomic annotations, providing an end-to-end solution for automated high-throughput single-cell genome reconstruction.

**Availability:**

CoSAG-nf is freely available under the MIT License at: https://github.com/linfengxu/CoSAG-nf. Archival code repository snapshots are published at zenodo with doi: https://doi.org/10.5281/zenodo.21525244.

## 1 Introduction

Single-cell genomics enables access to microbiome analyses, particularly powerful for resolving low-abundance taxa, strain-level variation, and novel biosynthetic pathways (Lasken and McLean 2014, Ide *et al*. 2022, Hosokawa and Nishikawa 2024). Advances in microfluidic sorting now routinely generate hundreds to thousands of single amplified genomes (SAGs) per study (Zheng *et al*. 2022, Ling *et al*. 2024, Zhang *et al*. 2025), but individual SAGs suffer from severe limitations. Amplifying the single copy of genomic DNA from an individual cell via multiple displacement amplification (MDA) introduces significant biases, resulting in uneven coverage, high contamination rates, and leaving a substantial proportion of the genome unsequenced (Gawad *et al*. 2016, Sobol and Kaster 2023). This leads to highly fragmented assemblies with median completeness often falling below 30%, limiting the utility of individual SAGs due to poor genome uniformity and completeness (Bowers *et al*. 2024). Co-assembly of related SAGs addresses this limitation by pooling reads from phylogenetically similar cells, leveraging complementary coverage to improve completeness from ∼40% to >85% or even from 17–25% to >90% in high-throughput applications (Kogawa *et al*. 2018, Zheng *et al*. 2022).

The ccSAG workflow (Kogawa *et al*. 2018) first demonstrated the feasibility of cross-reference cleaning and co-assembly of closely related SAGs, and several recent studies have further applied co-assembly strategies to specific datasets (Arikawa *et al*. 2021, Ide *et al*. 2022, Zhang *et al*. 2025). However, these efforts were typically implemented as case-specific analyses or collections of scripts, and to our knowledge no automated, containerized pipeline currently extends this approach to the upstream grouping and large-scale processing of thousands of low-completeness SAGs generated by modern droplet-based platforms. Researchers therefore still need to manually combine (1) similarity-based SAG grouping, (2) iterative contamination removal, (3) quality assessment, and (4) HPC parallelization into a coherent workflow. Such manual processes are error-prone and difficult to reproduce, particularly at the scale of modern high-throughput datasets.

To address this gap, we developed CoSAG-nf, an automated, end-to-end Nextflow pipeline for SAG co-assembly at scale, implemented following nf-core framework standards (Ewels *et al*. 2020). Here, we describe the pipeline architecture and optimization algorithm, and validate its performance through a three-tiered evaluation: (i) a four-species mock community for benchmarking species assignment accuracy and assembly quality against ground truth; (ii) 7984 human saliva SAGs for evaluating performance on a complex real-world dataset; and (iii) a human gut SAG dataset for cross-validation against a previously published manually curated co-assembly.

## 2 Methods

### 2.1 Pipeline overview and design

**Figure 1:**
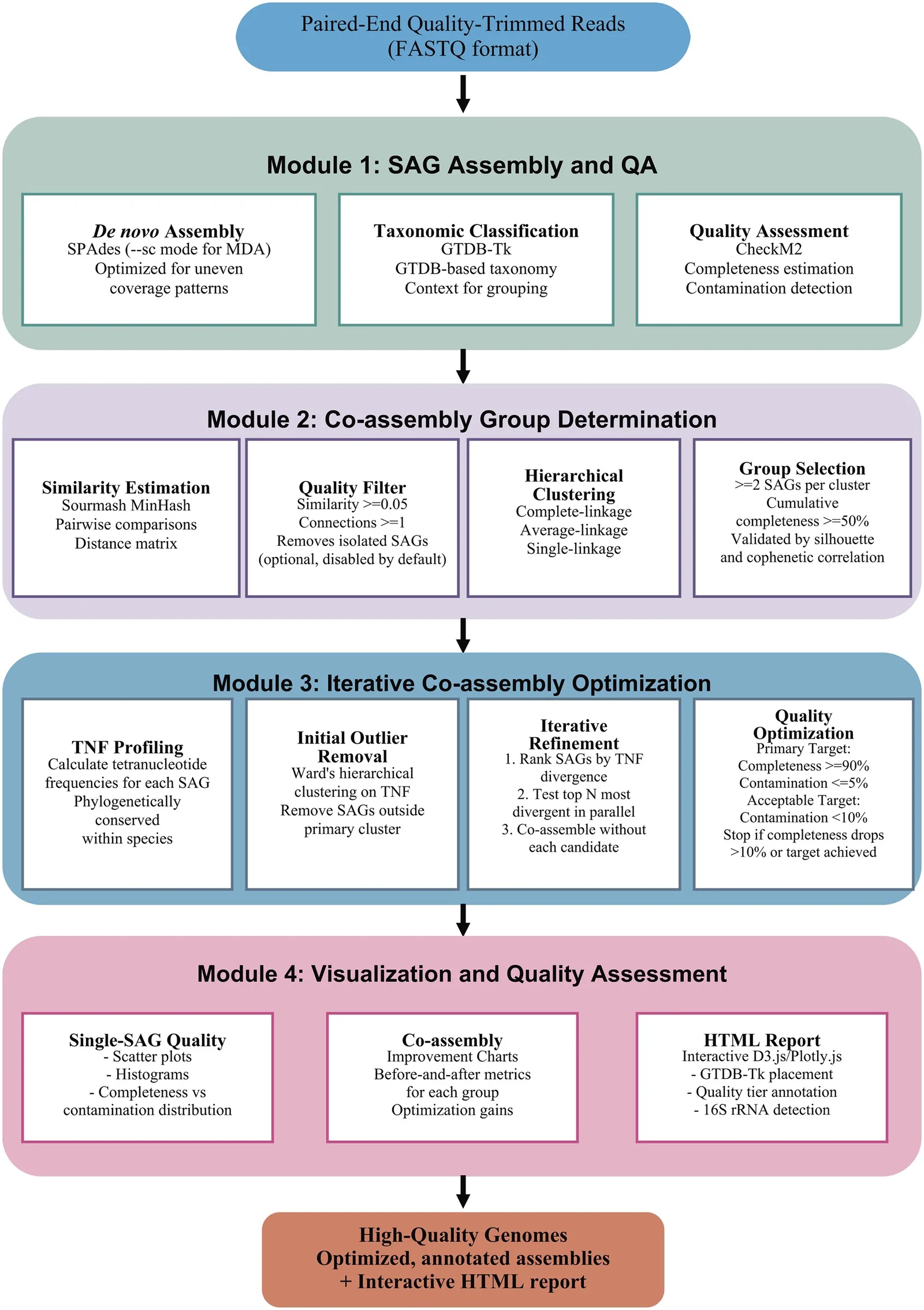
CoSAG-nf workflow for single-cell genome co-assembly and optimization. The pipeline consists of four integrated modules: (1) Individual SAG Assembly—de novo assembly and initial quality assessment of single-cell amplified genomes; (2) Clustering for Co-assembly Groups—alignment-free, genome similarity-based grouping using sourmash MinHash signatures; (3) Iterative Optimization—iterative removal of tetranucleotide-frequency outlier SAGs followed by re-assembly; (4) Quality Reporting—generation of interactive HTML reports with comprehensive visualization of assembly statistics and taxonomic assignments.

CoSAG-nf is a Nextflow-based computational pipeline for reconstructing high-quality microbial genomes from SAG sequencing data (Fig. 1). The workflow comprises four interconnected modules that automate genome assembly, quality assessment, and optimization:

1. Individual SAG Assembly and Quality Assessment: *De novo* assembly of clean sequencing reads with comprehensive quality assessment
2. SAG Grouping and Clustering: Alignment-free genomic comparison to identify putatively conspecific SAGs
3. Iterative Co-assembly Optimization: Systematic refinement of co-assemblies through contamination-aware SAG selection
4. Results Visualization and Reporting: Generation of interactive HTML reports with integrated quality metrics and phylogenetic context

### 2.2 Module 1: single-cell amplified genome assembly and quality assessment

The pipeline accepts pre-processed paired-end sequencing reads in FASTQ format as input. Input files are assumed to be demultiplexed (individual FASTQ pairs for each SAG), quality-trimmed, and, where relevant, host-depleted prior to execution. De novo assembly is performed using SPAdes (Bankevich *et al*. 2012) with the –sc flag, which implements specialized algorithms to handle the characteristically uneven read coverage resulting from multiple displacement amplification (MDA). Assembly quality is evaluated using CheckM2 (Chklovski *et al*. 2023), which estimates genome completeness and contamination using a machine learning model trained on annotated reference genomes. SAGs exceeding a user-configurable contamination threshold (–max_contamination, default 10%) are filtered at this stage. Taxonomic classification is performed using GTDB-Tk (Chaumeil *et al*. 2022) to provide biological context for the processed SAGs for end users only and does not influence downstream processing, which relies on annotation-agnostic k-mer similarity clustering.

### 2.3 Module 2: identification of co-assembly groups through genome clustering

This module employs a multi-stage clustering approach to identify groups of related SAGs amenable to co-assembly. Pairwise genomic similarity is estimated using sourmash (Brown and Irber 2016), which generates MinHash sketches enabling rapid, alignment-free comparison of all qualifying SAGs.

The resulting sourmash MinHash Jaccard similarity matrix is converted to a distance matrix and subjected to agglomerative hierarchical clustering. Multiple linkage methods are supported with the SciPy Python package (Virtanen *et al*. 2020) including complete-linkage (which maximizes minimum intra-cluster similarity), average-linkage, and single-linkage, providing flexibility for different dataset characteristics. Clustering quality is assessed using silhouette coefficients and cophenetic correlation, and groups are delineated under user-configurable cut parameters. Although prokaryotic species are conventionally delineated at ≥95% average nucleotide identity (ANI), this value is used here only as a biological reference for interpreting putatively conspecific groups, rather than as an operational threshold applied during clustering, which operates on MinHash Jaccard distances (Jarett *et al*. 2018, Nowinski *et al*. 2023). Within each group, all non-singleton SAGs are then co-assembled into a single composite assembly; singletons are retained as-is. This first-pass co-assembly is performed for every non-singleton group, independently of whether the group subsequently enters the Module 3 optimization step. Each co-assembly is then evaluated with CheckM2. Co-assemblies that meet the completeness threshold but exceed the contamination threshold enter the Module 3 iterative optimization step, in which the most TNF-divergent SAGs are iteratively removed; all others are retained as-is. We refer to each retained co-assembled product as a co-assembled SAG (CoSAG), that is, either the direct output of Module 2 (without optimization) or the output of Module 3 after removal of the most TNF-divergent SAGs (with optimization).

### 2.4 Module 3: iterative optimization of co-assemblies using tetranucleotide frequency profiling

Module 3 is conditionally activated for candidate CoSAGs from Module 2 that combine high completeness with elevated contamination, a profile indicative of a near-complete co-assembly burdened by a small number of divergent SAGs. In such cases, elevated CheckM2 contamination does not necessarily indicate exogenous sequences. Rather, it reflects the presence of related yet genomically divergent lineages, whose heterogeneous signals resemble the multi-genome admixture patterns that CheckM2 was trained to flag as contamination. Briefly, CheckM2 estimates contamination using a gradient-boosted model trained on protein-annotation features from synthetic (including artificially contaminated) genomes, rather than the single-copy marker-gene redundancy used by CheckM1 (Parks *et al*. 2015, Chklovski *et al*. 2023).

To resolve this, Module 3 exploits tetranucleotide frequency (TNF) profiles, compositionally conserved genomic signatures that carry phylogenetic signal and are shared among closely related organisms while diverging between taxonomically distinct ones (Pride *et al*. 2003), to detect intra-cluster genomic heterogeneity. Jaccard distance is unsuitable here because it defines the grouping itself and is confounded by partial genome recovery, whereas TNF is compositional, requires no shared sequence between two SAGs, and operates at the taxonomic resolution at which the outliers actually differ.

Initial outlier detection applies Ward’s hierarchical clustering on Euclidean distances between per-SAG TNF profiles within the candidate CoSAG, identifying the dominant compositional subgroup and provisionally excluding the remaining, divergent SAGs. This initial step provides a coarse separation only; final membership is determined by the subsequent quality-driven refinement. The algorithm then enters an iterative refinement phase: in each iteration, the three most TNF-divergent SAGs are selected as removal candidates and evaluated through parallel trial co-assemblies.

**Figure 2:**
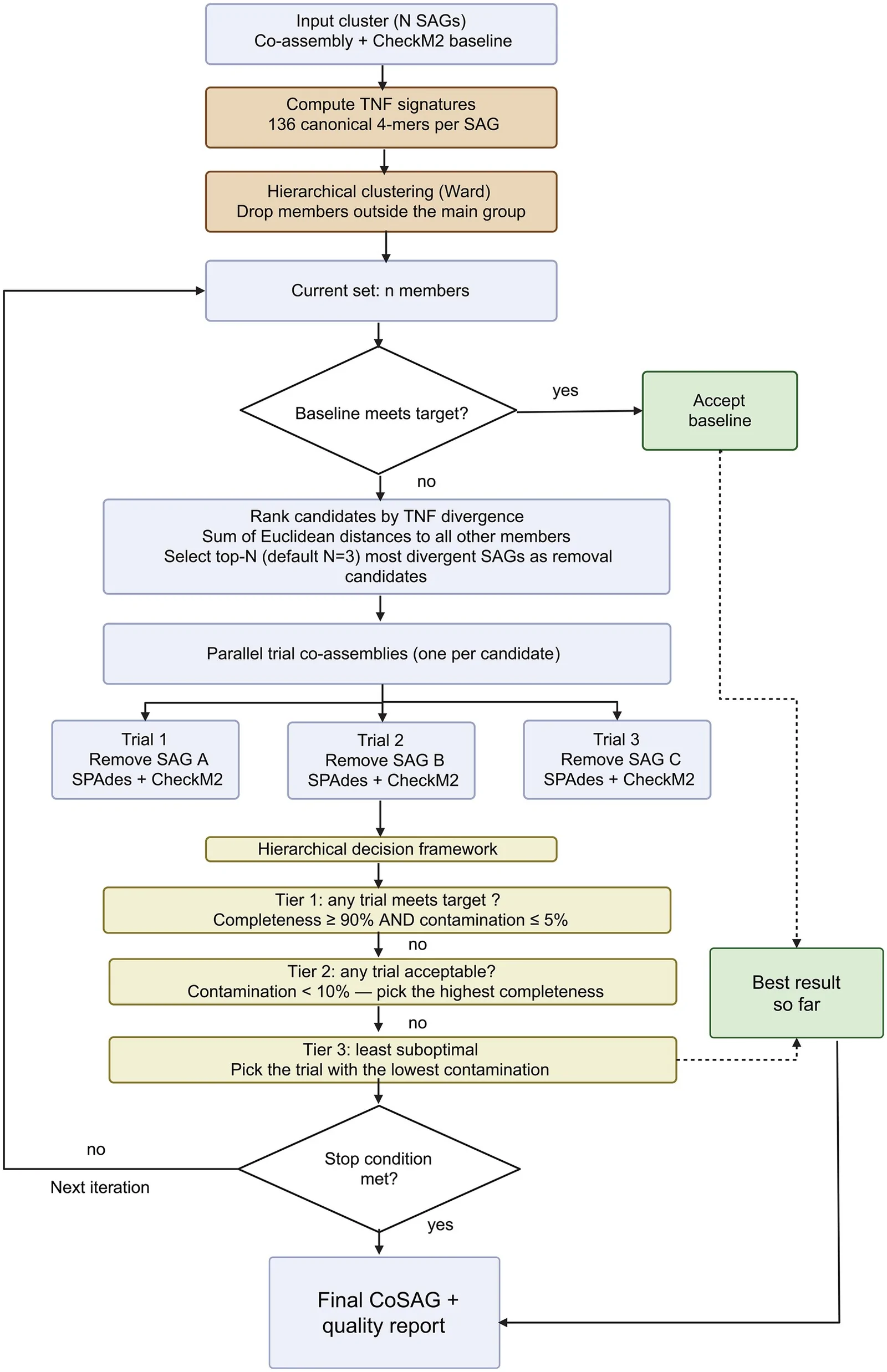
Quality-gated iterative co-assembly refinement in CoSAG-nf: starting from a TNF-filtered cluster, the most TNF-divergent SAGs are iteratively removed via parallel trial co-assemblies, with each round selected by a tiered completeness/contamination decision framework until a stop condition is met.

Optimization terminates when the Tier-1 target is reached, when completeness drops sharply between iterations (indicating over-optimization), or when the cluster reaches predefined floors on completeness, member count, or iteration number. All activation, ranking, and termination thresholds are exposed as configurable parameters, with defaults summarized in Fig. 2.

### 2.5 Module 4: interactive visualization and comprehensive quality reporting

The final module generates self-contained, interactive HTML reports summarizing all assembly results and quality metrics. The reporting framework processes JSON-formatted (Pezoa *et al*. 2016) hierarchical data structures containing SAG quality statistics, co-assembly configurations, and taxonomic classifications.

Key analytical features include: (i) automated genome quality stratification into high (Completeness > 90% and Contamination < 5%), medium (Completeness ≥ 50% and Contamination < 10%), and low (Completeness < 50%) categories following the MISAG standard (Bowers *et al*. 2017). Ribosomal RNA genes (5S, 16S and 23S) are additionally predicted for each CoSAG using barrnap v0.9, and the number of recovered copies is reported per genome. Because many bacterial genomes carry multiple rRNA operons whose intragenomic sequence divergence is typically below 1% (Pan *et al*. 2023), the recovery of 16S copies differing beyond this level within a single CoSAG provides an orthogonal indication of residual heterogeneity, complementary to marker-gene-based contamination estimates. Because contamination is increasingly underestimated at low completeness for any genome, both individual SAGs and co-assembled CoSAGs are considered reliable at low completeness only when contamination is also very low. The corresponding thresholds are user-configurable at both the SAG filtering and CoSAG stratification stages, allowing stricter contamination limits to be applied to low-completeness genomes where needed; (ii) dynamic, interactive visualizations implemented using Chart.js and Plotly.js libraries (JavaScript graphing library), including quality distributions, clustering efficiency metrics, contamination reduction analyses, and taxonomic diversity representations; (iii) tabbed navigation with real-time filtering, search functionality, and CSV export capabilities. The embedded data architecture eliminates external dependencies, ensuring report portability and long-term accessibility.

Comparative statistics quantifying pre- and post-optimization improvements in completeness and contamination enable users to assess the efficacy of the iterative refinement process. Together, these visualization and reporting capabilities transform complex multi-dimensional genomic data into accessible, structured outputs that facilitate scientific interpretation and support downstream analytical workflows.

### 2.6 Implementation and availability

The CoSAG-nf pipeline is implemented in Nextflow DSL2 following the nf-core framework, ensuring scalability, portability, and reproducibility through standardized workflow structure and containerized execution.

## 3 Results

### 3.1 Mock community benchmark validates the taxonomic fidelity and assembly quality of CoSAG-nf

We first benchmarked CoSAG-nf against a published SAG mock community (PRJNA803937; 5497 SAGs from *Escherichia coli*, *Klebsiella pneumoniae*, *Bacillus subtilis* and *Staphylococcus aureus*) (Zheng *et al*. 2022), whose complete reference genomes provide ground truth for both species’ assignment and assembly quality.

Individually assembled, 95.7% of SAGs had completeness <10% and 98.2% contamination <2% (Fig. S1, available as supplementary data at *Bioinformatics* online), and GTDB-Tk assigned taxonomy to only 19 SAGs (0.35%), reflecting insufficient marker-gene recovery for per-SAG placement (Chaumeil *et al*. 2022). Co-assembly is therefore a prerequisite for taxonomic analysis of such datasets.

The complete CoSAG-nf workflow recovered 45 CoSAGs meeting medium-quality or higher standards (CheckM2 completeness ≥50%, contamination <10%) from the 5497 input SAGs. CheckM2 completeness ranged from 50.2% to 100% (median 67.8%), contamination from 0.3% to 6.7% (median 3.4%), and genome sizes from 2.15 to 5.35 Mb (Fig. S2, available as supplementary data at *Bioinformatics* online). Of these, 10 CoSAGs reached high-quality status (completeness >90% and contamination <5%), while the remaining 35 were classified as medium-quality. A full overview of the CoSAG-nf outputs for the mock community dataset is provided in Supplementary Section 1.1, available as supplementary data at *Bioinformatics* online.

**Figure 3:**
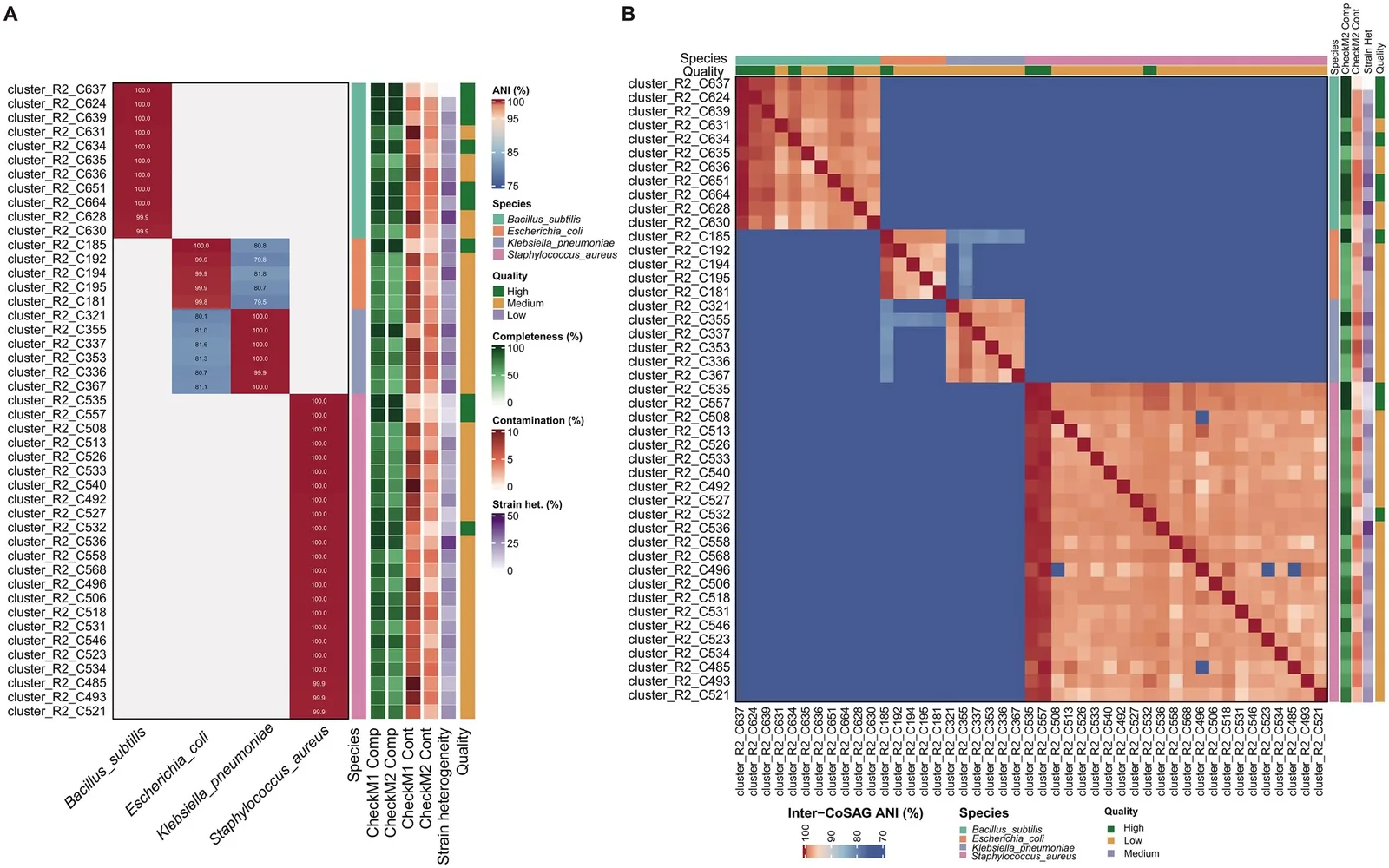
Benchmarking CoSAG-nf on a mock community dataset with known reference genomes. (A) Rows: 45 CoSAGs, grouped by FastANI species assignment (color bar). Left columns: pairwise FastANI values (%) between each CoSAG and the four reference genomes. Right columns: CheckM1/CheckM2 completeness and contamination, CheckM1 strain heterogeneity, and MIMAG quality tier. All 45 CoSAGs map uniquely to a single reference at ≥99.8% ANI. (B) Pairwise FastANI matrix among the 45 CoSAGs (rows and columns share the same order; species color bars on top and right). Single-linkage clustering at the 95% ANI species threshold yields exactly four species-level clusters, each corresponding precisely to one reference strain.

To evaluate species-assignment accuracy, each of the 45 CoSAGs was independently classified by whole-genome ANI and by 16S rRNA gene analysis. FastANI v1.34 (Jain *et al*. 2018) comparisons against the four reference genomes showed all 45 CoSAGs uniquely mapping to a single reference at 99.80%–100.00% best-hit ANI (Fig. 3A). For CoSAGs derived from *E. coli* and *K. pneumoniae*, ANI to the alternative Enterobacteriaceae reference fell within the expected inter-genus background range of 79.18%–81.77%, well below the 95% species threshold. Single-linkage clustering of the pairwise FastANI matrix at 95% ANI partitioned the 45 CoSAGs into exactly four species-level clusters matching the reference composition (*E. coli*, 5; *K. pneumoniae*, 6; *B. subtilis*, 11; *S. aureus*, 23). Among the 253 intra-cluster *S. aureus* pairs, one fell marginally below the threshold (C493–C558, 94.68%) and two (C485–C496 and C496–C508) yielded too few alignable fragments for an ANI estimate (Fig. 3B), most likely reflecting MDA coverage heterogeneity (Zhang *et al*. 2015). Nevertheless, the CoSAGs involved all retained ≥99.8% ANI to the *S. aureus* reference and were correctly grouped through single-linkage transitivity. No cross-species pair reached 95% ANI. The 16S rRNA gene analysis yielded fully concordant species assignments for all 45 CoSAGs, and seven CoSAGs recovered multiple rrn operon copies of the expected species, indicating that CoSAG-nf preserves intra-genomic sequence diversity during single-cell aggregation (Supplementary Section 1.2, available as supplementary data at *Bioinformatics* online). As an additional robustness check, completeness and contamination estimates obtained with CheckM v1.2.3 (Parks *et al*. 2015) were closely concordant with the CheckM2 values used throughout this study (Fig. 3B).

### 3.2 CoSAG-nf recovers high-quality genomes and resolves closely related taxa in a human saliva microbiome

We then applied the pipeline to 7984 SAGs generated from human saliva by Mozhuo Biotech (Zhejiang) Co., Ltd. following a previously described protocol (Zheng *et al*. 2022); detailed sample collection and sequencing procedures are provided in Supplementary Sections 2.1–2.3, available as supplementary data at *Bioinformatics* online.

**Figure 4:**
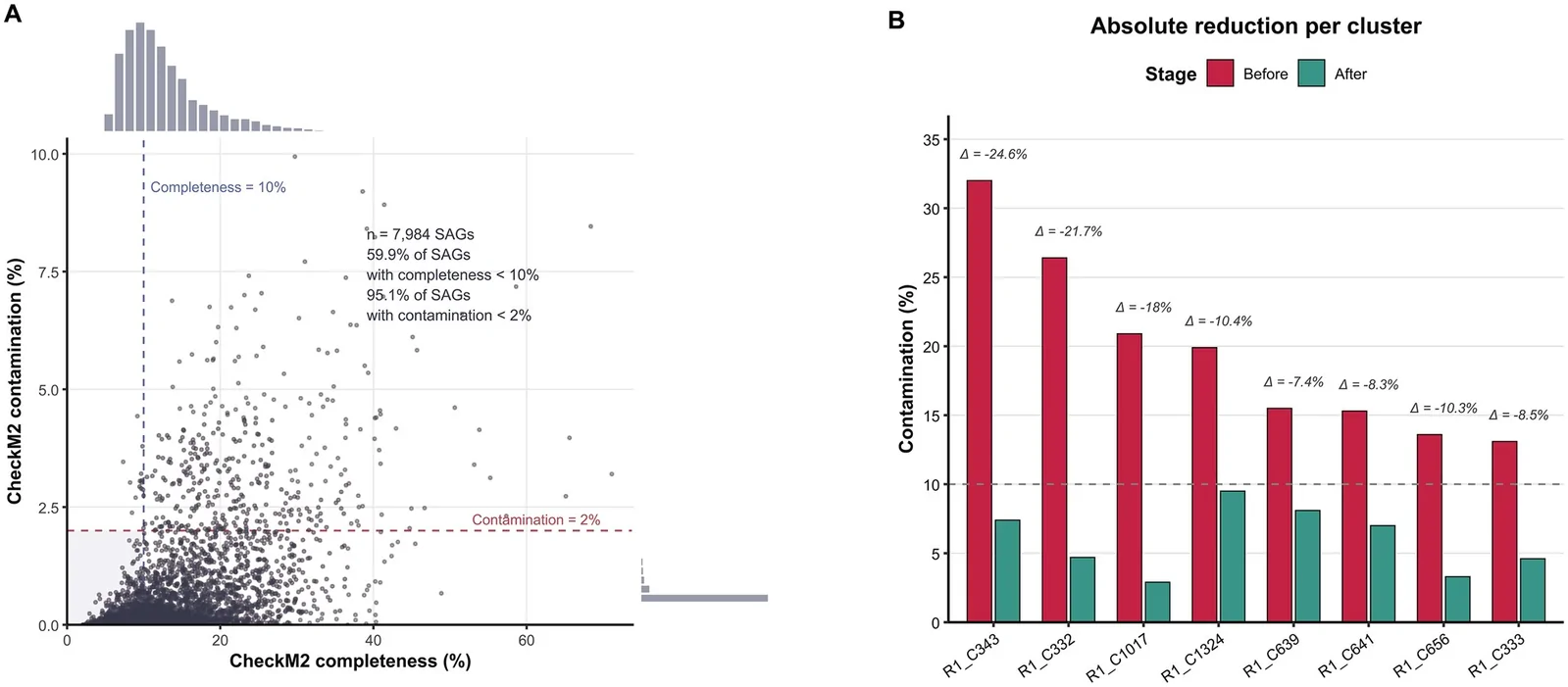
Baseline SAG quality and TNF-based decontamination in the saliva dataset. (A) Baseline quality assessment of 7984 single-cell amplified genomes (SAGs) from saliva community by CheckM2. (B) Per-cluster contamination before and after co-assembly optimization. Δ Indicates absolute reduction (percentage points).

As in the mock community, the individual SAGs were highly fragmented (median completeness 8.7%, range 2.0%–71.1%; median contamination 0%; Fig. 4A), and only 11 of the 7984 SAGs (0.14%) met medium-quality standards on their own. CoSAG-nf assigned 1227 of the 7984 SAGs (15.4%) to clusters that yielded co-assemblies passing quality control, generating 180 CoSAGs comprising 5 high-quality genomes (CheckM2 completeness >90%, contamination <5%) and 175 medium-quality genomes (completeness ≥50%, contamination <10%). The remaining SAGs either fell below the minimum cluster size required for co-assembly or produced co-assemblies that did not meet medium-quality criteria.

Among the high-quality assemblies, CoSAG-nf recovered near-complete genomes for several key saliva-associated taxa, including *Fusobacterium periodonticum*_B (95.2% completeness, 4.7% contamination), *Fusobacterium pseudoperiodonticum* (91.6% completeness, 4.6% contamination), and *Streptococcus mitis*_BP (93.0% completeness, 2.4% contamination). Two further high-quality CoSAGs fell below the 95% species threshold against unnamed GTDB placeholders (*Streptococcus* sp013277115, 94.7%; *Granulicatella* sp947254045, 92.2%), consistent with candidate novel oral taxa. The benefit of the Module 3 optimization step was most apparent in this dataset. A subset of co-assembled clusters showed high completeness but residual contamination ≥10%, triggering the TNF-based iterative optimization module (Module 3). Across these clusters, contamination was reduced from 13.1%–32.0% before optimization to 2.9%–9.5% afterwards (Fig. 4B). For example, contamination in the *Fusobacterium periodonticum*_B cluster C332 fell from 26.4% to 4.7% while completeness was retained at 95.2%. No cluster in the mock community met the trigger criteria, consistent with the low contamination of that dataset.

CoSAG-nf also resolved closely related species within complex species groups. Within the *Streptococcus mitis* species complex, the pipeline recovered distinct CoSAGs assigned to multiple GTDB species clusters (*n* = 5, completeness 51.2%–93.0%, contamination 1.4%–9.9%). Similar resolution was observed for the *Streptococcus oralis* group (*n* = 3, completeness 56.8%–75.5%, contamination 0.7%–8.6%) and for the *Fusobacterium periodonticum* complex as delineated in GTDB (*n* = 3, completeness 50.1%–95.2%, contamination 1.7%–9.5%). These results highlight the capacity of CoSAG-nf to capture fine-scale taxonomic diversity that is typically collapsed under broader species definitions in NCBI-based taxonomy.

Applied to a saliva community, CoSAG-nf therefore recovered 180 genome-resolved units spanning multiple closely related species within the *S. mitis* and *F. periodonticum* complexes, together with two candidate novel taxa, illustrating its utility for fine-scale taxonomic characterization of complex oral communities (Supplementary Section 2.4, available as supplementary data at *Bioinformatics* online).

### 3.3 Application to a human gut sample reproduces a manually curated co-assembly workflow

To evaluate the practical utility of CoSAG-nf on real human microbiome samples, we applied the pipeline to a single gut single-cell sample (sample #4, 7,019 SAGs) from project PRJNA803937. Although the mock community benchmark and this gut sample originate from the same project, they represent two distinct data types: the mock community is an artificial assemblage of four predefined reference strains used to validate methodological accuracy, whereas the gut sample is a real microbial community of unknown composition that potentially contains uncultured taxa, used here to evaluate co-assembly performance in a realistic and complex setting.

As in the saliva dataset, the individual SAGs were highly fragmented: 6807 of the 7019 SAGs (96.9%) had completeness below 10%, and 7004 (99.8%) had contamination below 2%. The complete CoSAG-nf workflow recovered 68 CoSAGs meeting MIMAG medium-quality or higher standards, of which 7 reached high quality (completeness >90%, contamination <5%) and the remaining 61 were medium quality. CheckM2 completeness ranged from 50.6% to 100% (median 65.0%), contamination from 0.5% to 9.9% (median 3.9%), and genome size from 1.44 to 5.24 Mb (Fig. S3, available as supplementary data at *Bioinformatics* online).

GTDB-Tk classification assigned the 68 CoSAGs to 5 phyla, 5 classes, 7 orders, 9 families, and 31 genera. Bacillota_A predominated (88.2%, 60/68), followed by Bacteroidota (7.4%, 5/68), with single CoSAGs assigned to Bacillota_C, Bacillota_I, and Actinomycetota. Three CoSAGs carried a genus-level assignment but no species-level match in GTDB, representing potential species-level novel genomic resources.

**Figure 5:**
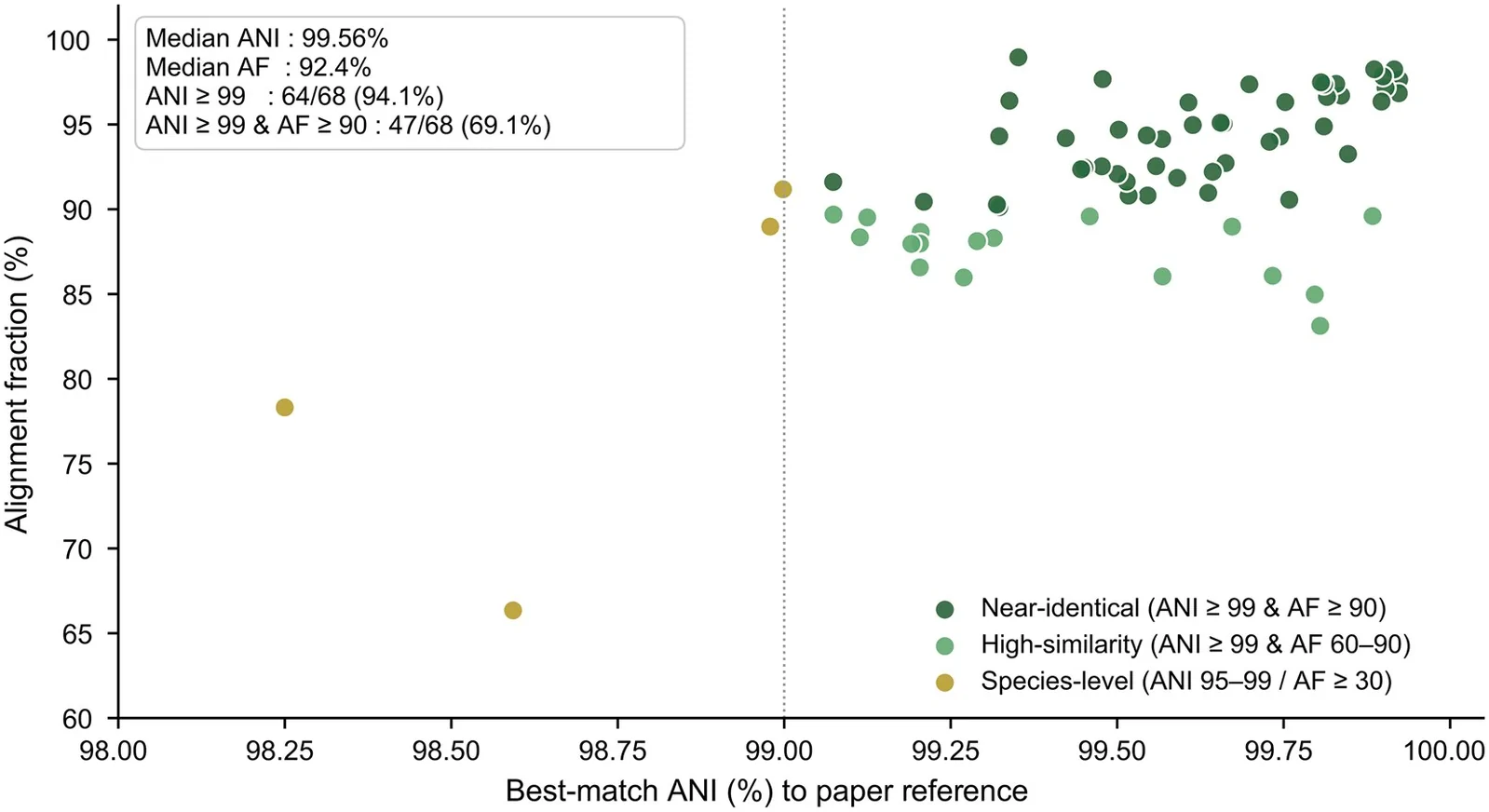
Species-level concordance between CoSAG-nf assemblies and the published reference set. Each point represents one of the 68 CoSAGs produced by CoSAG-nf from gut sample #4, plotted by best-match average nucleotide identity (ANI) and alignment fraction (AF) against the published reference set, computed with FastANI v1.34.

To assess consistency with previously published SAG co-assembly workflows, we used the co-assemblies reported by Zheng *et al.* as a reference benchmark (Zheng *et al*. 2022). That study applied custom scripts to the full PRJNA803937 dataset, yielding 76 CoSAGs at the consensus level (hereafter the published reference set). We compared each of the 68 CoSAGs produced by CoSAG-nf on the single sample against this reference set using FastANI v1.34, retaining the highest-ANI match for each. All 68 CoSAG-nf assemblies matched a reference genome at ≥98.25% best-hit ANI (median 99.56%, IQR 99.32%–99.77%) with a median alignment fraction of 92.4%, exceeding the 95% species delineation threshold in every case, and 64 (94.1%) reached ≥99% ANI (Fig. 5, Table S1, available as supplementary data at *Bioinformatics* online). Because this comparison uses a single sample rather than the full dataset, some low-abundance taxa present in the reference set were not expected to be recovered.

These results show that an automated, containerized workflow reproduces the species-level output of a manually curated co-assembly pipeline on the same data, while extending the analysis to a second body site with comparable genome quality.

### 3.4 Reproducible implementation, interactive reporting, and computational performance

**Figure 6:**
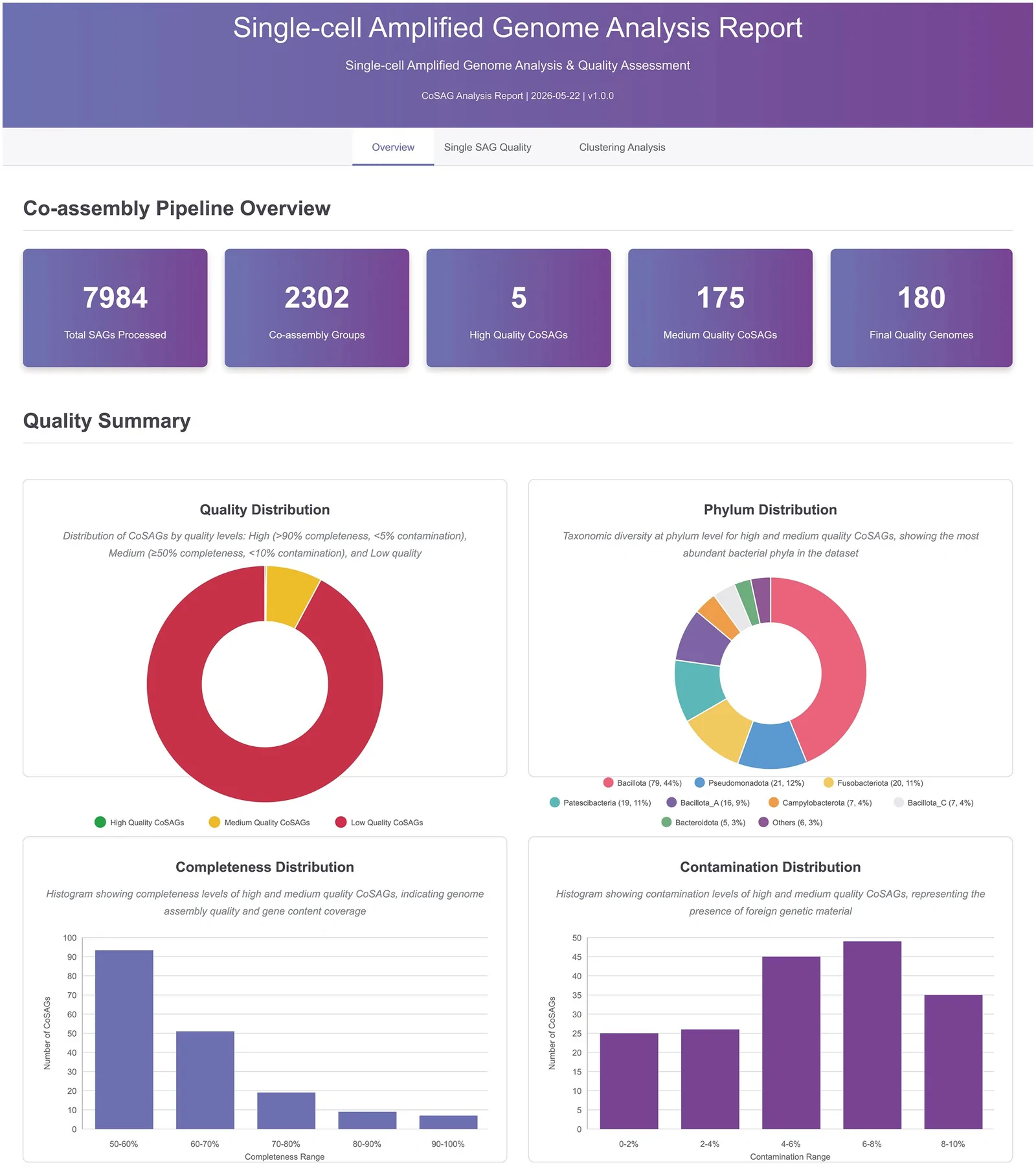
Overview of the CoSAG-nf pipeline on the saliva dataset.

Each CoSAG-nf run produces a self-contained interactive HTML report summarizing the analysis (Fig. 6, Figs S2–S3, available as supplementary data at *Bioinformatics* online). The report presents the quality distribution of the input SAGs and the resulting CoSAGs, the composition and taxonomic assignment of each cluster, and the pre- and post-optimization statistics for clusters processed by Module 3, with filtering by taxonomy, cluster size, and quality metrics. This allows users to inspect individual clustering decisions and to trace each CoSAG back to its constituent single cells without re-running the pipeline. Example reports for the mock, saliva and gut datasets are available at https://github.com/linfengxu/CoSAG-Reports.

CoSAG-nf is implemented in Nextflow with Docker and Singularity environment definitions for every process, so that the entire workflow runs from raw SAG reads to annotated CoSAGs with a single command and a fixed software stack. Runtime was benchmarked on three datasets of several thousand SAGs each, including a human gut dataset (7019 SAGs) comparable in scale to previously published work. All runs were executed on a single node (20 CPUs, 480 GB memory) and completed within 3.7–8.9 wall-clock hours (Table S2, available as supplementary data at *Bioinformatics* online), confirming that CoSAG-nf is feasible for datasets of thousands of SAGs on a single node. Because the per-SAG assembly and per-cluster co-assembly stages are independent, the workflow scales with the available cores and can be resumed after interruption without repeating completed steps.

## 4 Discussion

We present CoSAG-nf, a scalable Nextflow pipeline designed to address challenges in reconstructing high-quality microbial genomes from high-throughput single-cell genome sequencing datasets. As single-cell genomics technologies continue to generate increasingly large SAG collections from diverse samples, the demand for automated, computationally efficient methods to process hundreds to thousands of SAGs simultaneously has become essential.

Compared to existing pipelines such as ccSAG, CoSAG-nf offers several key advantages for high-throughput applications. While ccSAG employs iterative read-mapping to raw contigs for chimera detection and removal through cross-reference cleaning, this alignment-intensive strategy becomes computationally prohibitive when scaling to large SAG collections (Kogawa *et al*. 2018). CoSAG-nf instead leverages MinHash-based genome similarity estimation for rapid initial clustering, circumventing the need for extensive pairwise alignments during SAG grouping. This design prioritises scalability, and rather than performing dedicated chimera detection, CoSAG-nf reduces inter-species chimera risk indirectly through conservative clustering thresholds and taxonomic consistency checks.

Furthermore, ccSAG requires 16S rRNA similarity (≥99% in V3-V4 region) and ANI >95% for initial SAG classification, necessitating sufficient 16S rRNA gene recovery, a requirement that may not be met in incomplete SAGs (Alneberg *et al*. 2018, Arikawa *et al*. 2021). CoSAG-nf’s alignment-free MinHash approach operates effectively even with fragmented assemblies, providing more robust clustering for low-quality initial SAGs.

Recent high-throughput studies have adapted co-assembly concepts to larger scales. The workflow described by Zheng *et al*. (2022) in their human gut microbiome study processed thousands of SAGs using custom scripts for clustering and co-assembly, but this approach requires substantial bioinformatics expertise to implement and lacks standardized quality optimization. These manual workflows are not readily transferable between studies and offer limited reproducibility.

CoSAG-nf implements an intelligent, iterative refinement algorithm that systematically identifies and removes contaminating SAGs based on tetranucleotide frequency divergence, employing hierarchical quality targets and multi-criterion termination strategies to prevent over-optimization. This approach directly addresses the challenge of inter-SAG contamination within co-assembly groups, a source of quality degradation distinct from the intra-SAG chimeric sequences that ccSAG targets through read-level cleaning. By operating at the SAG-level rather than the read-level, CoSAG-nf’s optimization is computationally tractable for large datasets while achieving substantial contamination reduction. The parallelizable design enables simultaneous optimization of multiple SAG groups across computing clusters. Tractability comes from constraining the search space: rather than evaluating all possible exclusion combinations, each iteration tests only a small number of candidate co-assemblies, with each trial operating on a single small cluster of related SAGs.

The integrated visualization and reporting module transforms complex quality metrics, taxonomic classifications, and optimization outcomes into accessible, interactive HTML reports, facilitating rapid assessment across thousands of SAGs. This capability is essential for high-throughput projects, where manual curation is impractical. The self-contained reports ensure long-term accessibility and support reproducible research practices.

CoSAG-nf is subject to several methodological caveats. The upfront hierarchical clustering removes obvious outliers efficiently but may over-prune intrinsically homogeneous clusters, as the subsequent greedy refinement cannot reinstate prematurely excluded members. The TNF-based ranking is most sensitive to phylogenetically distant contamination; closely related contaminants may share similar tetranucleotide signatures with the target and require complementary chimera detection (e.g., GUNC) for reliable removal (Orakov *et al*. 2021). Relatedly, because the iterative refinement reduces contamination by removing divergent members, in a minority of clusters this can lower completeness, reflecting an inherent precision-completeness trade-off. Default parameters were calibrated on the mock community benchmark and are exposed via the command-line interface for application-specific tuning.

## 5 Conclusion

By providing an automated, scalable workflow for high-throughput SAG processing, CoSAG-nf addresses a practical bottleneck in large-scale single-cell microbial genomics. The pipeline allows researchers to recover usable population-level genomes from large single-cell sequencing datasets that would otherwise remain fragmented and below usable quality as individual SAGs, supporting the characterization of uncultivated microbial diversity in complex environmental and host-associated microbiomes.

## Supplementary Material

btag671_Supplementary_Data

## Data Availability

The datasets supporting the conclusions of this study are available as follows. Newly generated saliva SAG data have been deposited in the National Omics Data Encyclopedia (NODE; https://www.biosino.org/node/) under accession OEP00007126. Publicly available datasets used for benchmarking were obtained from NCBI SRA under accessions [PRJNA803937].
