# Supplementary material for: CoSAG-nf: a scalable nextflow pipeline for co-assembly, optimization, and interactive visualization of high-throughput single-cell genomes
Source: Bioinformatics. 2026 Sep 17;42(9):btag671. doi: 10.1093/bioinformatics/btag671 (PMC13614715; doi:10.1093/bioinformatics/btag671)
Supplement: Supplementary file 1 [file btag671_supplementary_data.zip › Supplementary Data.pdf]

Supplementary Data

CoSAG-nf: A Scalable Nextflow Pipeline for Co-assembly, Optimization, and Interactive Visualization of High-Throughput Single-Cell Genomes

Section 1

1.1 Overview of CoSAG-nf outputs

The recovery of 45 CoSAGs from four reference strains reflects a deliberate design trade-off in CoSAG-nf. To minimize the risk of inter-species chimerism caused by erroneous merging, the workflow adopts a relatively conservative distance threshold at the sourmash MinHash clustering stage (Module 2), which separates SAG subsets exhibiting substantial divergence in k-mer composition into distinct clusters (Ondov, et al., 2016; Pierce, et al., 2019). Because MDA amplification stochastically favours different genomic regions across cells, SAGs originating from the same strain frequently show heterogeneous coverage profiles (Hosokawa, et al., 2017; Kogawa, et al., 2018; Zhang, et al., 2015); at an input scale of 5,497 SAGs, the emergence of multiple independent CoSAGs per reference strain is therefore an expected outcome rather than an artifact. In downstream applications, users may merge closely related CoSAGs based on pairwise sourmash distances or FastANI comparisons to obtain species-level consensus genomes. In this study, we retained all 45 independent CoSAGs without further merging to fully showcase the sub-species- and strain-level resolution achievable by the pipeline.

A clarification regarding the TNF-based iterative optimization module (Module 3) is warranted. CoSAG-nf employs a conditionally triggered, modular design: Module 2 co-assembly products are first evaluated by CheckM2, and any candidate CoSAG falling within the completeness >90% and contamination >10% regime (default parameters, user-adjustable) automatically activates Module 3, which uses tetranucleotide-frequency (TNF) iterative optimization to remove outlier SAGs deviating from the cluster centroid and to re-assemble the refined set, thereby purifying near-complete but contamination-burdened CoSAGs to high-quality status. In the present mock community benchmark, none of the candidate CoSAGs produced by Module 2 fell within this

trigger regime, and Module 3 was consequently not activated. This outcome indicates that for datasets of low taxonomic complexity with well-separated reference taxa, the clustering and initial co-assembly performed by Module 2 alone are sufficient to yield CoSAGs meeting MIMAG quality standards (Bowers, et al., 2017). The empirical performance of Module 3 is systematically evaluated on a more challenging real-world dataset.

1.2 Accuracy of taxonomic assignment: dual validation by 16S rRNA

16S sequences were extracted with barrnap (v0.9, --kingdom bac) and aligned by BLASTN to a custom reference comprising the multi-copy 16S genes of the four mock-community strains (Altschul, et al., 1990; Seemann, 2013). Every CoSAG yielded at least one classifiable 16S sequence, with primary-hit alignment lengths of 635–1,549 bp (median 1,547 bp; 35/45 ≥1,400 bp, 41/45 ≥1,000 bp) and identities of 99.87–100.00%. In all 45 CoSAGs the 16S primary-hit species matched the FastANI assignment, yielding 100% (45/45) concordance between marker-gene and whole-genome taxonomy.

Multi-fragment 16S recovery occurred in 11 CoSAGs and resolved cleanly into two categories: expected multi-operon recovery and artefactual signal. (i) Seven CoSAGs (*K. pneumoniae*: C321, C336, C353, C355; *B. subtilis*: C628, C634, C635) recovered additional fragments mapping to different rrn operon copies of the same species (e.g., C336 recovered *K. pneumoniae* 16S copies 4, 6 and 8 at 100% identity). Because bacterial genomes typically carry 5–10 16S rRNA copies that diverge slightly across operons, these recoveries reflect faithful multi-operon assembly from a single cell and provide additional evidence of CoSAG completeness (Acinas, et al., 2004; Větrovský and Baldrian, 2013). (ii) Four CoSAGs (C518, C557, C624, C639) carried short cross-species secondary hits (430–502 bp), in every case residing on low-coverage contigs (2.99–10.34×) clearly distinguishable from the corresponding high-coverage primary 16S contigs (35.2–169.6×; 8.6- to 56.7-fold higher), with bitscores 2.4- to 3.6-fold below the on-target primary hits. This signature (short fragment, low coverage, sharply reduced bitscore) is most consistent with trace cross-well or reagent-derived background DNA introduced during MDA, a well-documented artefact of single-cell whole-genome amplification (Blainey and Quake, 2011; Woyke, et al., 2011), and did not affect species assignment in any case.

Together, two methodologically independent lines of evidence (whole-genome ANI and a conserved

single-copy marker gene) deliver 100% concordant species assignments for all 45 CoSAGs. The recovery of multiple rrn operon copies in 7 CoSAGs further demonstrates that CoSAG-nf preserves intra-genomic structural diversity during single-cell aggregation, while the small, low-coverage off-target signals observed in 4 CoSAGs are unambiguously distinguishable from genuine assembly content and traceable to known MDA artefacts. These results jointly establish the taxonomic fidelity of the CoSAG-nf workflow.

1.3 Cross-tool quality assessment with CheckM1 and CheckM2

We additionally report CheckM1 completeness (Parks, et al., 2015), contamination, and strain-heterogeneity estimates alongside the CheckM2 values used during in-pipeline filtering (Chklovski, et al., 2023) (**Figure 3A**). Across the 45 CoSAGs, CheckM1 and CheckM2 gave broadly consistent results for both completeness (Pearson r = 0.96) and contamination (Pearson r = 0.43, with CheckM2 returning slightly lower values on average). CheckM1 strain heterogeneity ranged from 0% to 40.0% (median 23.1%), with all values below the 50% threshold conventionally used by CheckM1 to flag high heterogeneity (Parks, et al., 2015). These results are consistent with the species-level taxonomic concordance established above.

1.4 Summary

Taken together, the mock-community evaluation supports the performance of CoSAG-nf across three dimensions of CoSAG quality. First, taxonomic assignment was accurate at both the whole-genome and marker-gene levels: every CoSAG mapped uniquely to a single reference strain at 99.80–100.00% best-hit ANI (FastANI), and the 16S rRNA primary-hit species matched the FastANI assignment for all 45 CoSAGs (100% concordance). Second, assembly quality met community standards: all 45 CoSAGs satisfied MIMAG medium-quality criteria, with 10 reaching high-quality. Third, CheckM1 and CheckM2 gave broadly consistent quality estimates, and CheckM1 strain heterogeneity remained below the 50% threshold for all CoSAGs, providing no signal inconsistent with the species-level taxonomic assignments. These conclusions draw on multiple tools (FastANI, barrnap with mock-community 16S references, CheckM1, CheckM2) that operate on largely distinct features of the assembled sequences.

Notably, all 45 CoSAGs in this benchmark were produced by Module 2 (sourmash MinHash

clustering followed by co-assembly) without triggering Module 3 under default conditions, indicating that for simple communities with well-separated species, the Module 2 workflow alone is sufficient to recover medium- to high-quality CoSAGs. The TNF-based refinement in Module 3 is designed as a contamination-purification step for more complex scenarios involving high species diversity and closely related taxa; its performance on such samples is examined in the following sections.

Section 2. Saliva microbiome SAG dataset: sample collection, sequencing, and pipeline performance

2.1 Sample collection and pooling

Saliva samples were collected from 15 healthy volunteers and pooled prior to single-cell sequencing in order to broaden microbial diversity coverage and minimize the influence of inter-individual variation (Lazarevic, et al., 2010; Zhu, et al., 2020). Stored samples (−80 °C, 25% glycerol) were thawed on ice, resuspended, and sequentially filtered through 50 μm and 10 μm nylon membranes to remove host cells and large debris. Filtrates were concentrated by centrifugation and combined at equal cell counts (normalized by OD) to form a single pooled microbial suspension.

2.2 Single-cell library preparation and sequencing

The pooled microbial suspension was processed at Mozhuo Biotech (Zhejiang) Co., Ltd., using a droplet-based single-cell whole-genome sequencing protocol previously described (Zheng, et al., 2022). In brief, the workflow included viability assessment by Live/Dead staining, droplet encapsulation and lysis, multiple displacement amplification (MDA) for whole-genome amplification, intra-droplet tagmentation and barcoding, droplet breakage and bead-based library purification, and Illumina paired-end sequencing (Zheng, et al., 2022).

2.3 Sequencing summary and SAG yield

Sequencing of the pooled saliva sample produced approximately 510 million raw reads (153 Gbp total bases) and yielded 7,984 single-cell amplified genomes (SAGs) after barcode demultiplexing and quality filtering. Summary statistics are provided in **Table S3.**

2.4 Summary

The co-assembly approach substantially improved genome quality by pooling multiple low-quality SAGs. Across the five high-quality CoSAGs, individual SAGs were highly fragmented and incomplete (average completeness 18.1-23.5%), but co-assembly recovered near-complete genomes (91.1-95.2% completeness) while keeping contamination low (2.4-4.9%), representing 4.05- to 5.25-fold gains in completeness.

For example, *Streptococcus mitis_BP* improved from 9 low-quality SAGs (average completeness: 20.6%, average contamination: 0.69%) to a single high-quality genome (completeness: 93.0%, contamination: 2.4%), a 4.51-fold improvement in completeness. Similarly, an unclassified *Streptococcus* (R1_C882) improved from 13 SAGs (average completeness: 18.1%, average contamination: 0.25%) to a high-quality genome (completeness: 94.8%, contamination: 4.9%), a 5.25-fold improvement.

These results demonstrate that no single SAG approached the quality threshold on its own; only by co-assembling reads across cells of the same population could complete, low-contamination genomes be recovered, underscoring the necessity of co-assembly for SAG-based genome recovery.

Supplementary Figure

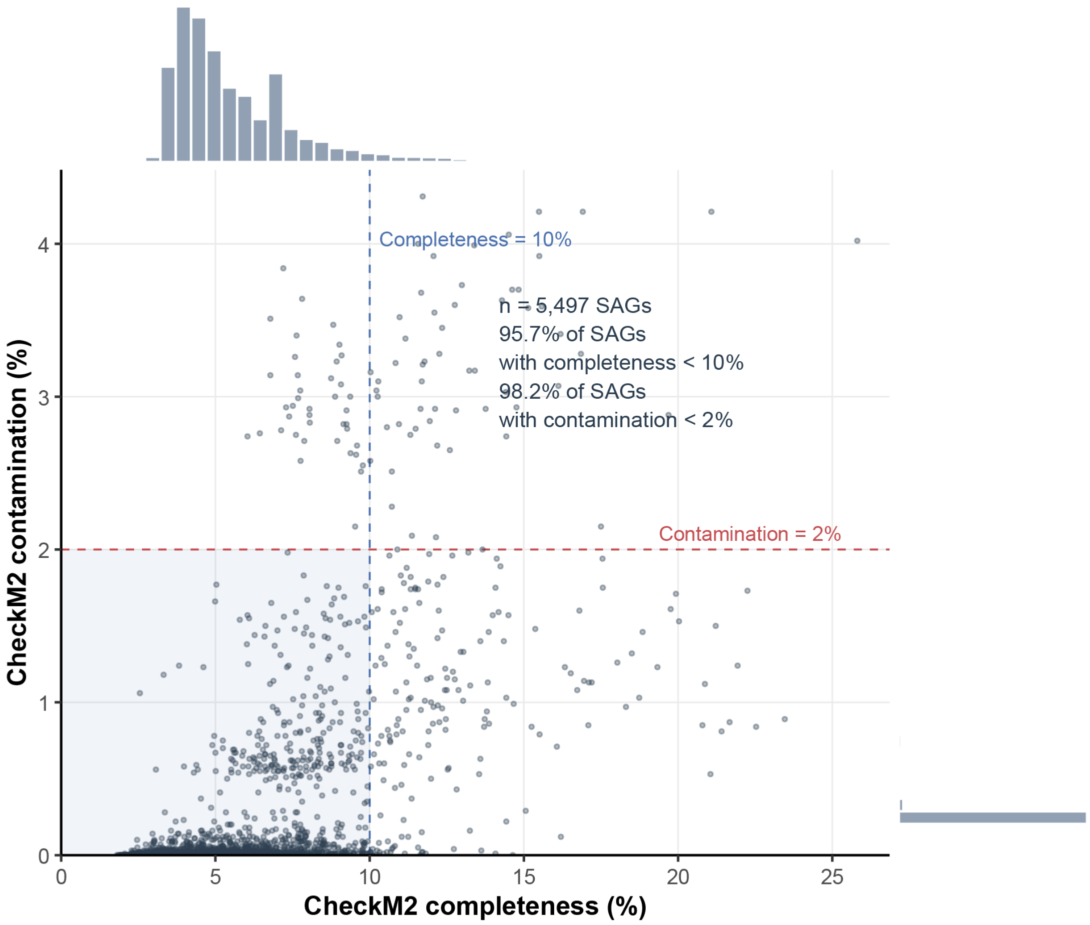

Figure S1. Baseline quality assessment of 5,497 single-cell amplified genomes (SAGs) from mock community by CheckM2.

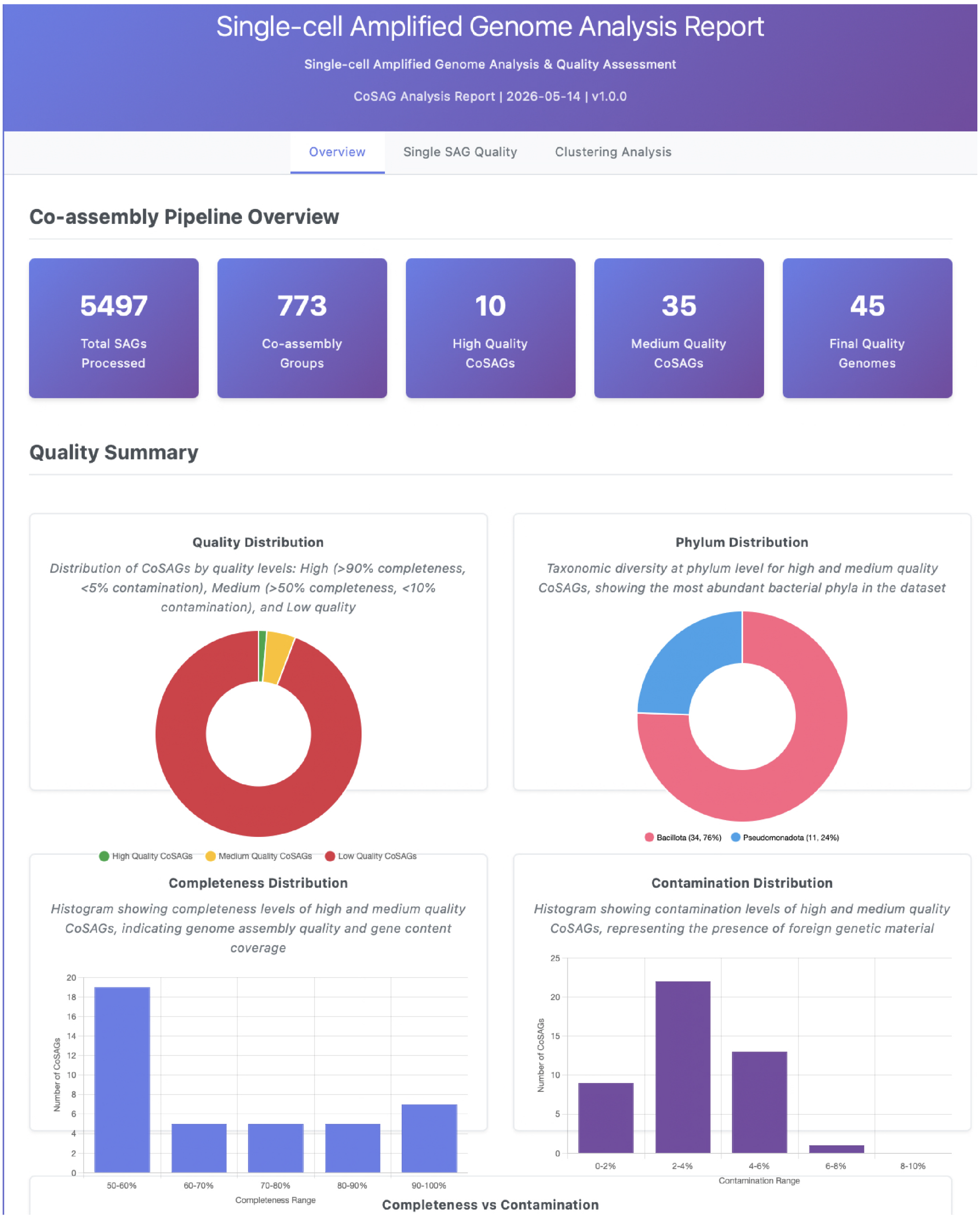

Figure S2. Overview of the CoSAG-nf pipeline on the mock community dataset

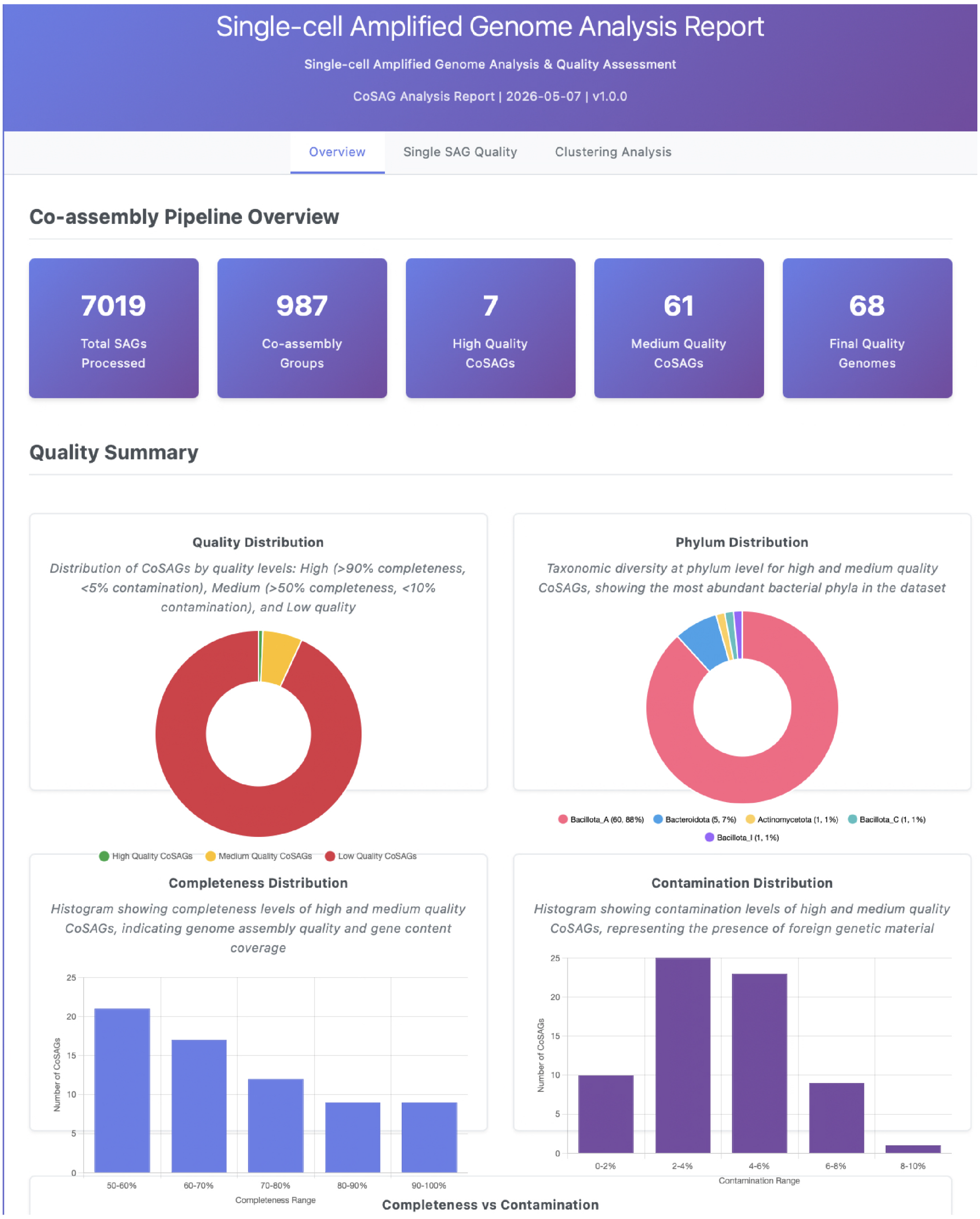

Figure S3. Overview of the CoSAG-nf pipeline on the gut dataset

Reference

Acinas, S.G., *et al.* Divergence and redundancy of 16S rRNA sequences in genomes with multiple rrn operons. *J Bacteriol* 2004;186(9):2629-2635.

Altschul, S.F., *et al.* Basic local alignment search tool. *J Mol Biol* 1990;215(3):403-410.

Blainey, P.C. and Quake, S.R. Digital MDA for enumeration of total nucleic acid contamination. *Nucleic Acids Res* 2011;39(4):e19.

Bowers, R.M., *et al.* Minimum information about a single amplified genome (MISAG) and a metagenome-assembled genome (MIMAG) of bacteria and archaea. *Nat Biotechnol* 2017;35(8):725-731.

Chklovski, A., *et al.* CheckM2: a rapid, scalable and accurate tool for assessing microbial genome quality using machine learning. *Nat Methods* 2023;20(8):1203-1212.

Hosokawa, M., *et al.* Massively parallel whole genome amplification for single-cell sequencing using droplet microfluidics. *Sci Rep* 2017;7(1):5199.

Kogawa, M., *et al.* Obtaining high-quality draft genomes from uncultured microbes by cleaning and co-assembly of single-cell amplified genomes. *Sci Rep* 2018;8(1):2059.

Lazarevic, V., *et al.* Study of inter- and intra-individual variations in the salivary microbiota. *BMC Genomics* 2010;11:523.

Ondov, B.D., *et al.* Mash: fast genome and metagenome distance estimation using MinHash. *Genome Biol* 2016;17(1):132.

Parks, D.H., *et al.* CheckM: assessing the quality of microbial genomes recovered from isolates, single cells, and metagenomes. *Genome Res* 2015;25(7):1043-1055.

Pierce, N.T., *et al.* Large-scale sequence comparisons with sourmash. *F1000Res* 2019;8:1006.

Seemann, T. barrnap 0.9: rapid ribosomal RNA prediction. *Google Scholar* 2013;792.

Větrovský, T. and Baldrian, P. The variability of the 16S rRNA gene in bacterial genomes and its consequences for bacterial community analyses. *PLoS One* 2013;8(2):e57923.

Woyke, T., *et al.* Decontamination of MDA reagents for single cell whole genome amplification. *PLoS One* 2011;6(10):e26161.

Zhang, C.Z., *et al.* Calibrating genomic and allelic coverage bias in single-cell sequencing. *Nat Commun* 2015;6:6822.

Zheng, W., *et al.* High-throughput, single-microbe genomics with strain resolution, applied to a human gut microbiome. *Science* 2022;376(6597):eabm1483.

Zhu, C., *et al.* Intraindividual Variation and Personal Specificity of Salivary Microbiota. *J Dent Res* 2020;99(9):1062-1071.
